# In Memory of Edward Diener: Reflections on His Career, Contributions and the Science of Happiness

**DOI:** 10.3389/fpsyg.2021.706447

**Published:** 2021-05-14

**Authors:** Weiting Ng, William Tov, Ruut Veenhoven, Sebastiaan Rothmann, Maria José Chambel, Sufen Chen, Matthew L. Cole, Chiara Consiglio, Arianna Costantini, Jesus Alfonso Daep Datu, Zelda Di Blasi, Susana Llorens Gumbau, Alexandra Huber, Saskia M. Kelders, Jeff Klibert, Hans Henrik Knoop, Claude-Hélène Mayer, Mirna Nel, Marisa Salanova, Marijke Schotanus-Dijkstra, Rebecca Shankland, Akihito Shimazu, Peter M. ten Klooster, Maria Vera, Maria A. J. Zondervan-Zwijnenburg, Llewellyn Ellardus van Zyl

**Affiliations:** ^1^School of Humanities and Behavioural Sciences, Singapore University of Social Sciences, Singapore, Singapore; ^2^School of Social Sciences, Singapore Management University, Singapore, Singapore; ^3^Erasmus Happiness Economics Research Organization, Erasmus University Rotterdam, Rotterdam, Netherlands; ^4^Optentia Research Focus Area, North-West University (VTC), Vanderbijlpark, South Africa; ^5^Faculdade de Psicologia, Universidade de Lisboa, CicPsi, Portugal; ^6^Graduate Institute of Digital Learning and Education, National Taiwan University of Science and Technology, Taipei, Taiwan; ^7^College of Business and Information Technology, Lawrence Technological University, Southfield, MI, United States; ^8^Department of Psychology, Sapienza University of Rome, Rome, Italy; ^9^Department of Human Sciences, University of Verona, Verona, Italy; ^10^Department of Special Education and Counselling and Integrated Centre for Wellbeing, The Education University of Hong Kong, Pok Fu Lam, Hong Kong; ^11^School of Applied Psychology, University College Cork, Cork, Ireland; ^12^WANT Research Team, Universitat Jaume I, Castelló de la Plana, Spain; ^13^Department of Medical Psychology, Medical University of Innsbruck, Innsbruck, Austria; ^14^Centre for eHealth and Wellbeing Research, University of Twente, Enschede, Netherlands; ^15^Department of Psychology, Georgia Southern University, Statesboro, GA, United States; ^16^Danish School of Education - General Education, Aarhus University, Aarhus, Denmark; ^17^Department of Industrial Psychology and People Management, University of Johannesburg, Johannesburg, South Africa; ^18^DIPHE, Department of Psychology, Education and Vulnerabilities, Université Lumière Lyon 2, Lyon, France; ^19^Faculty of Policy Management, Keio University, Endo, Fujisawa, Japan; ^20^Department of Education and Social Psychology, Pablo de Olavide University, Seville, Spain; ^21^Department of Methodology and Statistics, Utrecht University, Utrecht, Netherlands; ^22^Department of Industrial Engineering, University of Eindhoven, Eindhoven, Netherlands; ^23^Department of Human Resource Management, University of Twente, Enschede, Netherlands; ^24^Department of Social Psychology, Institut Für Psychologie, Goethe University, Frankfurt am Main, Germany

**Keywords:** Ed Diener, subjective well-being, life satisfaction, happiness, positive psychology, obituary, obituary announcement

## Introduction

Prof. Edward (Ed) Diener (1946-2021), a pioneer in positive psychology, passed away on the 27th of April 2021 at his home in Salt Lake City, Utah (Salt Lake City Tribune, 2021). As one of the most influential psychologists of the discipline, Ed Diener pushed the boundaries of our understanding of positive psychological functioning, subjective well-being, and happiness (Layous, 2020). As one of the Top 200 most cited researchers across all disciplines and fields, he will be most remembered for founding the scientific study of subjective well-being (SWB) and happiness (Bakshi, 2019). Diener developed the concept of subjective well-being by exploring the factors that influence people's life satisfaction (Diener et al., 2017a). He studied the individual causes of subjective well-being, such as close social relationships, income, meaning and purpose, personality, and societal causes, such as economic development, low corruption and crime, and a healthy environment (Diener et al., 2018). His research has discovered both universal and culture-specific causes and consequences of SWB and influenced governmental policy (Oishi et al., 1999). In respect of his memory, the purpose of this paper is threefold: (a) to reflect upon his career journey, (b) to celebrate his significant contributions to the discipline, and (c) to provide personal reflections of those who worked closely with him over the past 50 years.

## Career and Interests

When his academic career began as a Ph.D. student at the University of Washington (1970-1974), Diener's primary Research Topic was not happiness but rather deindividuation and its effects on aggression and transgressive behaviour (Diener, 2008; Layous, 2020). It would not be until many years later that his first seminal contribution to the study of subjective well-being would be published (Diener, 1984; Bakshi, 2019). Despite showing interest in ‘the positive’ during his Ph.D. years, the prevalent opinion at the time that happiness could not (or should not) be studied scientifically caused Diener to pursue more “acceptable” Research Topics (Layous, 2020). In 1974, Diener accepted an assistant professor position at the University of Illinois at Urbana-Champaign, where he would remain for most of his career (Diener, 2008). In 1979, he was promoted to a tenured associate professor and only then did he truly feel “free to begin studying happiness” (Diener, 2008, p. 4). In the years following, Diener, along with his many students and collaborators, would map out much of the terrain of well-being science, setting the stage for what is now known as “Positive Psychology” (Layous, 2020).

In addition to being a prolific researcher, Diener had a deep desire to help the world (Diener, 2008). He spearheaded the drive to create national accounts of well-being for policy purposes, believing that a focus on economic development alone was insufficient for ensuring a high quality of life (Diener, 2000; Diener and Seligman, 2004). Diener consulted with the United Nations and contributed to several reports on the value of well-being measures for public policy (Diener, 2008). His work helped build the empirical base that convinced governments in many countries to adopt well-being measures in their assessment of progress and quality of life (Bakshi, 2019). Diener also cared deeply about the broader discipline of psychology. He initiated the Noba project to provide free, online educational materials for the teaching of psychology—recruiting esteemed researchers to contribute reviews of their topic areas (Diener, 2008). Through Noba, Diener wanted to make psychology more accessible to a worldwide audience. He also hoped that students would learn more about recent advances in well-being science to benefit their own happiness. Drawing on the empirical literature, he and a team of psychologists developed the *ENHANCE* program (Enduring happiness and continued self-enhancement) —a series of lessons designed to teach social and coping skills for improving well-being (Heintzelman et al., 2020).

Diener also served the field in several leadership roles that aided in fast-tracking the establishment and growth of positive psychology as a discipline. He was appointed in 1999 as a senior advisor to the Gallup organisation in their attempts to investigate the functional importance of subjective well-being for society. He was also elected as the president of the International Society for Quality of Life Studies (1997-1998), Society for Personality and Social Psychology (2001), and the International Positive Psychology Association (2007-2009). Along with Ruut Veenhoven, he co-founded the *Journal of Happiness Studies* and acted as editor-in-chief for *Perspectives on Psychological Science*. His contributions have been recognized by the field several times. These include Distinguished Scientist Awards from the International Society of Quality of Life Studies (2000) and the American Psychological Association (2012), and the William James Lifetime Achievement award from the Association for Psychological Science (2013). He was an elected fellow of the American Academy of Arts and Sciences and was also a fellow of seven other scientific societies, including the American Psychological Association and the Association for Psychological Science. Of these many achievements, Diener remained very proud of helping to bring more attention to the scientific study of happiness and seeing young researchers take up the mantle and advance the field with rigorous, new methodologies (Diener, 2008).

## Key Contributions

### The Measurement of Well-Being

With over 400 scientific publications on human well-being and more than 257,000 citations, Diener was one of the most eminent psychologists in modern history (Layous, 2020). His seminal work on SWB, defined the construct as a multifaceted concept consisting of independent, separable components: life satisfaction—a cognitive, global evaluation of one's life, and the affective components—the frequency and intensity of positive and negative emotional experiences (Diener, 1984). Having a standard definition introduced greater consistency in the measurement and scientific study of SWB, amidst the available myriad of well-being scales. Diener made his own significant contributions to this area. The 5-item Satisfaction with Life Scale (Diener et al., 1985) has become the most widely used scale for assessing life satisfaction (Pavot and Diener, 1993; Van Zyl and Rothmann, 2014; Van Zyl et al., 2020). With a citation count exceeding 30 000, the Satisfaction with Life Scale has been translated into 39 different languages (Pavot and Diener, 1993; *https://eddiener.com**)*. In addition, Diener et al. developed the Scale of Positive and Negative Experience to measure positive and negative feelings (Diener et al., 2010a). They also developed a brief 8-item Flourishing Scale, which examines domains spanning social relationships, purpose, competence, self-esteem, and optimism, to assess psychological well-being (Diener et al., 2010a). Diener also utilized the experience sampling methodology to measure momentary affective states and minimize biases due to memory. One of the many important insights from this work was the importance of frequent rather than intense positive affect as a predictor of well-being (Diener et al., 1991). This work demonstrates his commitment to using multiple methods to study happiness and laid the foundation for building SWB focused process models.

### Building Process Models for SWB

Besides his pioneering work on the measurement of SWB, Diener's work encompasses areas examining the influences on SWB, outcomes of SWB, culture and SWB, and national accounts of SWB, with several influential papers published in these areas. For instance, research by Diener et al. has found that the factors influencing SWB relate differentially to the various SWB components (Diener et al., 2010b). They found that material wealth correlates more strongly with life satisfaction than positive or negative feelings, whereas psychosocial factors such as psychological needs (Diener et al., 2010c) and personality more strongly predict affective well-being. Their research further clarified the role of income in SWB. Their research showed that SWB increases with rising national income (Diener et al., 2013) and uncovered where the income satiation points were for different SWB components (Jebb et al., 2018). Other than examining the direct effects of causal factors, many of their research studies had delved further, to examine the mediating or moderating factors (e.g., national affluence, income inequality, perceived fairness) that may impact the relations between the predictors and SWB (e.g., Oishi et al., 2011).

### Well-Being and (Inter)National Policy

In his advocacy for well-being indicators in the policy arena, Diener made two important scientific contributions. First, the suitability of well-being as a policy target was sometimes questioned on the assumption that people adapt to all circumstances and experiences (Oishi and Diener, 2014). Diener et al. challenged this notion by showing that certain life events can have lasting effects on well-being (Diener et al., 2006). Second, to further convince policymakers of the value of well-being indicators, Diener began to redirect the attention of researchers and the general public from the causes of well-being to its *consequences*. Diener's work on the beneficial effects of happiness showed that happy people live longer and have superior health outcomes such as improved immune functioning and cardiovascular health (Diener and Chan, 2011; Diener et al., 2017b), are more productive and successful in work, more willing to help others, and have satisfying social relationships (Lyubomirsky et al., 2005). In short, happiness was good for society.

Diener et al. further highlighted societal and cultural differences in the frequency and intensity of positive and negative emotional experiences (Schimmack et al., 2002; Scollon et al., 2004). Differences in cognition (e.g., Asian cultures' dialectical way of thinking vs. Western cultures' analytical way of thinking), self-construal, and what is essential to happiness, was found to partly account for differences in SWB (Diener, 2008). Further, Diener (2008) established that there are both universal (e.g., social relationships, psychological needs) and culture-specific causes of SWB. For example, self-esteem correlates more strongly with life satisfaction in individualistic cultures than in collectivistic cultures (Diener and Diener, 1995). However, though current research indicates that Asian nations tend to report lower SWB than European American and Latin American nations, these societal differences may not only be due to psychological factors or values, but may largely be due to differences in objective circumstances between nations, as evident by findings that societal factors (e.g., national wealth, progressive taxation, income inequality, low corruption) account for substantial differences between nations (Jebb et al., 2020).

## Personal Reflections

### Ruut Veenhoven: Our Common Context

Printing seems to have been invented at different places around the same time. The same holds for the study of SWB. Independently, attempts to systematize concepts, measurement and research findings appeared in the late twentieth century. In the U.S., Diener published his programmatic paper on SWB in 1984 (Diener, 1984) and in the same year, I published my review of the then-available research findings on happiness (Veenhoven, 1984). In Australia, Bob Cummins started a similar program in the 1990s. We all gathered the then available stray studies on this subject in a Bibliography (Diener, 1984), Cummins (1997), of which mine is still maintained as part of the World Database of Happiness (Veenhoven, 2021).

Interest in SWB was in the air of the time, at least in western nations that experienced unprecedented peace and prosperity at the end of the twentieth century (Veenhoven, 2015). The existential security provided by society allowed individuals more choice in life, which made many people wonder how satisfying different ways of life will be for them. Factual knowledge about SWB was therefore welcomed and studies on SWB received much attention in the media.

However, the new strand of research met much scepticism in the scientific establishment of that time. In his autobiography, Ed Diener described an illustrative experience. As a student in the 1960s, he proposed doing a thesis on the happiness of migrant farmworkers, which seemed to him to be relatively happy even though relatively poor. He writes: “The professor was not pleased with my proposal. He said: “Mister Diener, you are not going to do this project for two reasons. First, I know that farmworkers are not happy and second, there is no way to measure happiness” (Diener, 2008, p.2). Some 15 years later, resistance against the study of SWB delayed his tenure at the University of Illinois. I had similar experiences, which has probably also happened to an unknown number of interested scholars who have dropped the subject for that reason.

Survivors of the initial academic resistance against the study of SWB picked fruits later, finding themselves in an open field with little competition. As Diener (2008) writes in his autobiography: “When I entered the field of subjective well-being, a few facts were already known. Nonetheless, most of the territory was uncharted” (p. 5). This allowed us to pioneer in answering a lot of highly relevant questions, such as whether greater happiness is possible in the human condition. Diener's unbridled energy enabled him to take these chances.

Diener's contribution to the field was not only in his own numerous publications but also in his scientific offspring. When research on SWB was no longer discouraged and basic questions settled, a new generation of interested young scholars presented itself. Diener had a good hand in selecting, training, and motivating these people, many of which contributed to the expansion of the field since 2000.

The development of a scientific specialization requires institutional settings that facilitate exchanging ideas, such as research associations and academic journals. Such a supportive environment was not found in the disciplinary organizations in the late twentieth century. Attention to SWB was still marginal on congresses and journal editors often rejected papers on the subject because they considered it unscientific. This pressed us to create our own settings. Together with Ed Diener and Alex Michalos, I founded the *Journal of Happiness Studies* in 2000, for which Diener functioned as an editor in chief in the years 2004-2006. We early birds were also involved in establishing the International Society for Quality of Life Studies (ISQOLS) in 1995 and in the International Positive Psychology Association (IPPA) in 2007.

The field of research on subjective well-being is now well-established. The next generation can build on the foundations laid by the first. Ed Diener is no longer among us, but his work lives on.

### Working With Ed: Personal Reflections From Will Tov and Weiting Ng

By the time we started graduate school in 2003 at the University of Illinois, Ed Diener was already a well-established researcher with over 125 journal articles, numerous other publications and serving as the editor of two journals. Positive psychology was still relatively young but Ed and his students had already uncovered a great deal of work and many fundamental findings on well-being. What could a young researcher tell Ed Diener about happiness that he did not already know? Yet, Ed possessed three traits that made it a great joy to work with him.

First, Ed was a genuinely positive person. This positivity manifested itself in countless ways. His voice often carried a sense of energy and engagement with life that was contagious. You could never fall asleep talking to Ed because he made anything he had to say sound exciting and important. He also had a wonderful mixture of kindness and humour. Once, Will brought a doughnut all the way from his parents' doughnut shop in California to Illinois for Ed to try. Before Will could give him advice about how long to heat it, Ed unwrapped it and ate it all up. “Tell your parents it was really good!” he said. When you are laughing at something Ed said or did, it's hard not to see him as anything other than a great human being.

Second, Ed was extremely down-to-earth. He never made his students feel like he was better than them, despite all of his many achievements. In fact, he often sought out their thoughts and opinions on various matters. He respected the intelligence of others, and if he felt that you might know something, he would ask you and really considered what you said. As a graduate student, this was very empowering. It was a tremendous boost of confidence to know that Ed Diener cared what you thought, and it made his students feel like they had a lot to offer to the field. If “subjective well-being” was the house that Ed built, his door was always open to others who wanted to come inside and help him build it.

Third, Ed had a strong sense of curiosity. He asked questions that were not always apparent but had important implications such as whether a person could be too happy, whether frequently thinking about one's happiness was a good thing or a bad thing, and whether happiness was beneficial in all cultures. Much of this curiosity fed his productivity as a researcher, and they were not always limited to questions about well-being. Ed thought a lot about the field of psychology as a whole—such as the career trajectory of eminent psychologists (Diener et al., 2014). Even if he had published a thousand papers, he still had ideas for another thousand. Thus, in Ed's eyes, there was always room for junior researchers to contribute.

## Conclusion

Ed Diener was a leading force in the development of the scientific study of subjective well-being and happiness. His work has been a fundamental building block on which the science of positive psychology and the careers of many researchers and psychologists are built. His ground-breaking ideas, work and legacy will continue to inspire our society to feel good, function well and flourish. His presence will be deeply missed.

## Post-Scriptum

For more information about Prof. Edward (Ed) Diener's life, career, contributions and work, please visit his personal website (*https://eddiener.com**)*.

## Author Contributions

LvZ conceptualized and finalized the first draft manuscript and attended to the revisions. WN, WT, RV, and SR took the lead in writing the first draft of the manuscript. The rest of the editorial board reviewed and commented on the final draft of the manuscript. All authors contributed to the article and approved the submitted version.

## Conflict of Interest

The authors declare that the research was conducted in the absence of any commercial or financial relationships that could be construed as a potential conflict of interest.
